# TLR2 Directing PD-L2 Expression Inhibit T Cells Response in *Schistosoma japonicum* Infection

**DOI:** 10.1371/journal.pone.0082480

**Published:** 2013-12-20

**Authors:** Yanan Gao, Lin Chen, Min Hou, Yingying Chen, Minjun Ji, Haiwei Wu, Guanling Wu

**Affiliations:** 1 Department of Pathogen Biology and Immunology, Nanjing Medical University, Nanjing, Jiangsu, China; 2 Jiangsu Province Key Laboratory of Modern Pathogen Biology, Nanjing, Jiangsu, China; 3 Center for International Health Research, Rhode Island Hospital, Providence, Rhode Island, United States of America; 4 Department of Pathogen Biology and Immunology, Nanjing University of Chinese Medicine, Nanjing, Jiangsu, China; Queensland Institute of Medical Research, Australia

## Abstract

Toll-like receptor 2 (TLR2) was shown to be an important immune receptor involved in the recognition of schistosome antigens, especially soluble egg antigen (SEA). In mice models with *Schistosoma japonicum* acute infection, we observed enhanced T cell-mediated immune responses in TLR2 knock out (TLR2^−/−^) mice compared with B6 mice. In *Schistosoma japonicum* chronic infection models, programmed death ligand 1 (PD-L1) and programmed death ligand 2 (PD-L2) expression as well as TLR2 expression gradually increased in B6 mice, while only PD-L2 expression significantly decreased in TLR2^−/−^ mice. Meanwhile, Programmed Death 1(PD-1) expression on CD4^+^T cells was down-regulated in TLR2^−/−^ mice after a large number of egg appeared. We also found that stimulation with schistosome antigens, especially SEA, could up-regulate PD-L2 expression on BMDCs in a TLR2-dependent manner *in vitro*. Schistosome antigens primed-BMDCs with impaired expression of TLR2 or PD-L2 could induce CD4^+^T cells to produce low level of IL-10 or high level of IFN-γ. Our results indicated that TLR2 signaling can direct PD-L2 expression on DCs, which binds to PD-1 mainly on CD4^+^T cells, to help inhibit T cells response in *Schistosoma japonicum* infection.

## Introduction

Innate immune responses are initiated through recognition of pathogen-associated molecular pattern (PAMP) by pattern-recognition receptors (PRRs) on the host cells. Activation of PRRs leads to an immediate response to infection and can profoundly influence the development of an adaptive immune response [1], [2]. Toll-like receptors (TLRs) are a class of membrane-bound pattern recognition receptors that are capable of identifying particular pathogen associated molecular patterns (PAMPs) and danger associated molecular patterns (DAMPs). Initially discovered in insects, the Toll receptor was determined to play a function in innate immune protection against fungal infections. Pursuit of Toll homologues revealed 12 members in mice and 10 members in humans [3], which can identify a wide range of ligands. TLRs are well known to be required for host defence against infections, including Gram-positive and -negative bacteria, fungi, viruses and parasites [3], [4]. Additionally, some TLRs also trigger negative immune response through binding some certain ligands, which might contribute to avoid excessive inflammation and develop chronic course of the disease, especially in the helminth infection. Schistosomiasis model is one of the most important models of chronic disease to investigate the interplay of immune response and parasites' pathogenicity in the host. During natural infection, schistosome cercariae penetrate the host and develop into schistosomula, which migrate to the portal vasculature to maturate, pair and lay eggs [5]. Host immune response switches from Th1 to a dominant Th2 response as the disease progresses [6]. So far, many studies about innate immune response during *Schistosoma mansoni* infection have been focused on egg antigens to illuminate how host developed a predominant Th2 response after egg deposition. A lipid fraction from *Schistosoma mansoni* eggs containing lysophosphatidylserine (lyso-PS) has been shown to induce dendritic cells (DCs) activation that promotes Th2 and regulatory T-cell development in a TLR2-dependent mechanism [7]. Lacto-N-fucopentaose III (LNFPIII), a synthetic copy of a schistosome egg glycan, has been shown to promote Th2 differentiation by DCs via a TLR4-dependent pathway [8]. These data suggested that the interactions of TLRs and excellular PAMPs from schistosome eggs can initiate the cascade pathway and direct Th2-biased immune responses so as to take part in the eggs-induced immunopathology.

Dendritic cells (DCs) act as important messengers between the innate and adaptive immunity. DCs are professional antigen-presenting cells (APCs) since the principal function of DCs is to present antigens, and only DCs have the ability to induce a primary immune response in resting naïve T lymphocytes. To perform this function, DCs are capable of capturing antigens, processing them, and presenting them on the cell surface along with appropriate costimulation molecules. DCs express different Toll-like receptors, such as TLR-2, -3, -4, and -7 *et al.* After encountering different natural ligands or specific PAMPs from pathogens for these TLRs, DCs become activated and mature into APCs that can secrete Th1 or Th2 cytokines and prime naive T cells for a proper immune response. The role of DCs in the induction of of T cell immunity has been well documented. DCs are vital in directing the final T helper cell responses (Th1 [9], Th2 [10], Th17 [11], or Treg [12] etc) through the communication between direct cell-to-cell contact, or via cytokines secretion. Generally, T cell activation requires T-cell receptor (TCR) signal and the costimulatory signals. An example of these constimulatory signals includes the interaction of dendritic cell B7 family membrane proteins (such as CD80, CD86) with CD28 present on lymphocytes to help T cell activation [13]. Actually, the maintenance of normal lymphocyte function requires the precise control between positive and negative costimulatory signals. For instance, activated DCs can also express programmed death ligand 1 (PD-L1) and programmed death ligand 2 (PD-L2), which are both considered co-inhibitory molecules due to their capacity to bind programmed death 1 (PD-1), which is expressed on activated T cells [14]–[16]. Engagement of PD-1 by its ligands PD-L1 or PD-L2 can transduce a negative signal that inhibits T cell proliferation, cytokine production, and cytolytic function [17].

Our previous studies [18] showed that TLR2 knockout mice infected with *Schistosoma japonicum* for 6 weeks displayed lower egg burden as well as the enhancement of T cell activation as assayed by Th1/Th2 cytokine secretion compared to wild-type mice. It strongly suggested that TLR2 as an important immune receptor was involved in recognition of the components of schistosome antigens, and played a negative role in regulating T cell activation. In this study, we further confirmed the increased numbers and function of CD4^+^ and CD8^+^ T cells in mice with deficiency of TLR2 with the infection of *Schistosoma japonicum*. As this disease progressed, the expression of a pair of coinhibitory molecules, PD-L2 on dendritic cells and PD-1 on CD4^+^ T cells were significantly lower in TLR2 knockout mice than that in wild-type mice. Furthermore, our results indicated that *in vitro* stimulation with schistosome antigens, especially soluble egg antigen (SEA) could significantly induce the upregulation of PD-L2 on DCs, which was dependent on TLR2 expression, and the interaction between PD-L2 with PD-1 on T cells might inhibit T cell-mediated immune response. So this study will contribute to better understand one of the multiple mechanisms about schistosome-induced immunosuppression in the host based on host-pathogen immune recognition.

## Results

### TLR2^−/−^ mice infected with *S. japonicum* manifested enhanced T cell immune response

#### The percentages of CD4+T and CD8+T cell subsets of TLR2−/− mice were higher than those in B6 mice

**Figure 1:**
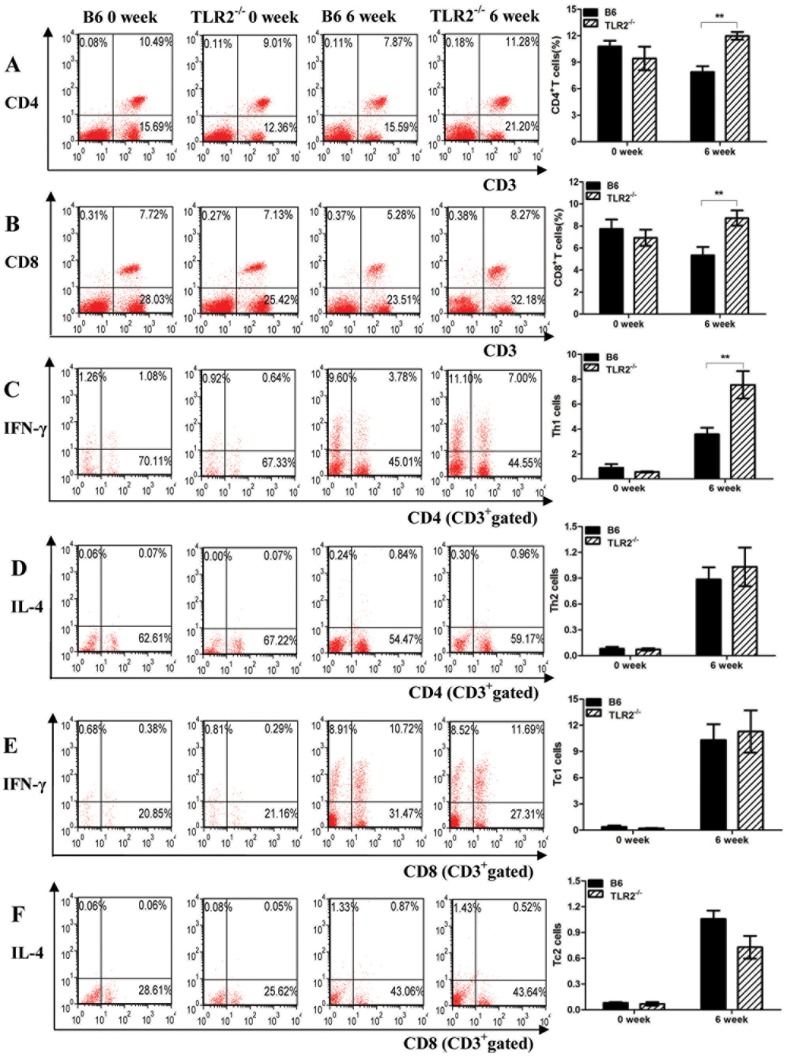
The percentages of CD4^+^T (A) and CD8^+^T (B) cells subsets in the splenocytes and the percentages of Th1(C), Th2(D), Tc1(E) and Tc2(F) in T lymphocytes in the spleens of TLR2^−/−^ and B6 mice at 0, 6 weeks post-infection with *Schistosoma japonicum* for 40 cercariae. Two independent infection experiments showed the similar results and here was the result of one experiment (*n* = 8). Data are presented as the mean±SEM. **P<0.01.

TLR2^−/−^ mice and wild-type (WT) mice were infected with 40 *Schistosoma japonicum* cercariae for 6 weeks. When these mice were sacrificed, TLR2^−/−^ mice presented less egg burden in the liver compared with WT mice as our previous study showed. At the same time, we observed the proportion of CD4^+^T (CD3^+^CD4^+^) and CD8^+^T (CD3^+^CD8^+^) cells in spleens of TLR2^−/−^ and B6 mice by flow cytometry. As shown in Figure 1, there were no significant differences in splenic CD4^+^T and CD8^+^T cells of TLR2^−/−^ mice compared to B6 mice before infection. At 6 weeks after *S. japonicum* infection, the percentage of CD4^+^T and CD8^+^T cells in TLR2^−/−^ mice was significantly higher than that in B6 mice. We further investigated the proportion of Th1 cells (CD3^+^CD4^+^IFN-γ^+^), Th2 cells (CD3^+^CD4^+^IL-4^+^), Tc1 cells (CD3^+^CD8^+^IFN-γ^+^) and Tc2 cells (CD3^+^CD8^+^IL-4^+^) in CD3^+^ T lymphocytes in the spleens of TLR2^−/−^ and B6 mice. Before infection, TLR2 knockout did not affect the development of Th1, Th2, Tc1, Tc2 cell subsets. We found that at 6 weeks after *S. japonicum* infection, the percentage of Th1 cells in TLR2^−/−^ mice was significantly more than that in B6 mice. No obvious differences were exhibited in the percentage of Th2, Tc1 and Tc2 between TLR2^−/−^ and B6 mice.

#### The transcription of some cytokine and cytotoxicity-related genes were elevated in T cells of TLR2−/− mice

**Figure 2:**
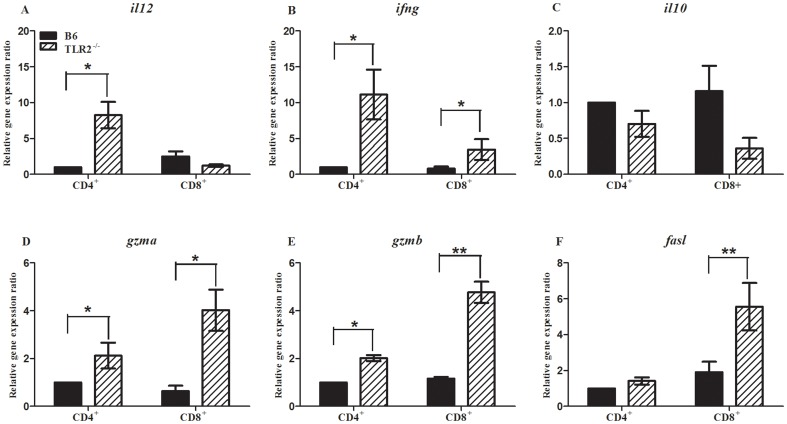
The relative mRNA levels of *il2*(A), *ifng*(B), *il10*(C), *gzma*(D), *gzmb*(E) and *fasl*(F) expression on isolated CD4^+^T and CD8^+^T cells in TLR2^−/−^ and B6 mice infected with *Schistosoma japonicum* for 40 cercariae. Two independent infection experiments showed the similar results and here was the result of one experiment (*n* = 8). Expression level for each gene was normalized to β-actin. Data are presented as the mean±SEM. *P<0.05, **P<0.01.

To evaluate the function of CD4^+^T and CD8^+^T cells in the absence of TLR2, CD4^+^T and CD8^+^T cells isolated with MACS from schistosome-infected TLR2^−/−^ and B6 mice were used to observe the transcription of some cytokine genes and cytotoxicity-related genes by qRT-PCR. At 6 weeks after *S. japonicum* infection, the transcription of *il12* and *ifng* in CD4^+^T cells, and the transcription of *ifng* in CD8^+^T cells were significantly elevated compared to those in B6 mice as shown in Figure 2. Moreover, the increment of *il12* and *ifng'*s transcription is more obvious in CD4^+^T cells than that in CD8^+^T cells. Besides these, TLR2^−/−^ mice also displayed the enhanced expression of *gzma* and *gzmb* in CD4^+^T cells, and *gzma*, *gzmb* and *fasl* in CD8^+^T cells compared to B6 mice. There was in no doubt that CD8^+^T cells presented more significant change in the expression of these cytotoxic genes.

### The deficiency of TLR2 mainly alleviated the expression of PD-L2, an important coinhibitory molecule

#### TLR2 expression was persistently upregulated in B6 mice after S. japonicum infection

**Figure 3:**
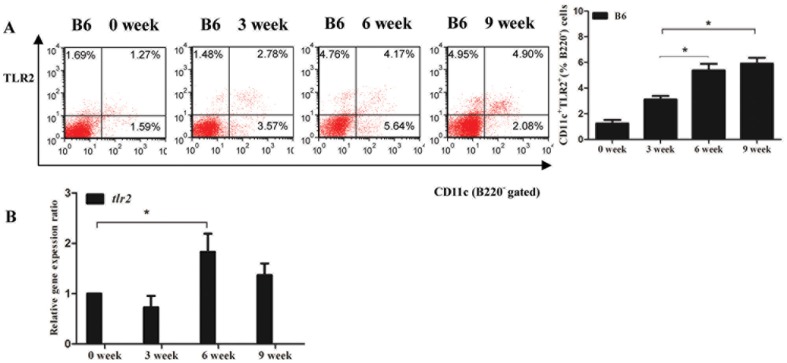
Dynamic changes of TLR2 expression(A) and mRNA transcription of *tlr2*(B) on CD11c^+^ cells from B6 mice during the chronic infection with *Schistosoma japonicum* for 14 cercariae. Here was the result of one experiment (n = 6). Data are presented as the mean±SEM. *P<0.05.

From the above experiments, the deficiency of TLR2 did enhance the host's T cells responses in schistosome acute infection, suggested that TLR2 might play a negative role in regulating immune reactions. Next, we set up the chronic infection model with 14 cercariae invasion to ascertain the dynamic characteristic of TLR2 expression in dendritic cells. Dendritic cells was labelled with CD11c^+^B220^−^ to exclude B cells contamination. As shown in Figure 3A, the expression of TLR2 on CD11c^+^ cells in spleen was gradually increased *in vivo* chronic infection with *S. japonicum* by flow cytometry analysis, especially after the onset of a large number of eggs. The expression of TLR2 on CD11c^+^ cells at 6 weeks and 9 weeks was significantly increased than that at 3 weeks. Then the mRNA levels of *tlr2* from CD11c^+^ cells in spleen were evaluated by qRT-PCR (Figure 3B). The transcription of *tlr2* in CD11c^+^ cells was significantly elevated at 6 weeks post-infection.

#### The expression of coinhibitory molecules, PD-L1 and PD-L2, was increased in B6 mice during schistosome infection, however, only PD-L2 expression was significantly reduced in the absence of TLR2

**Figure 4:**
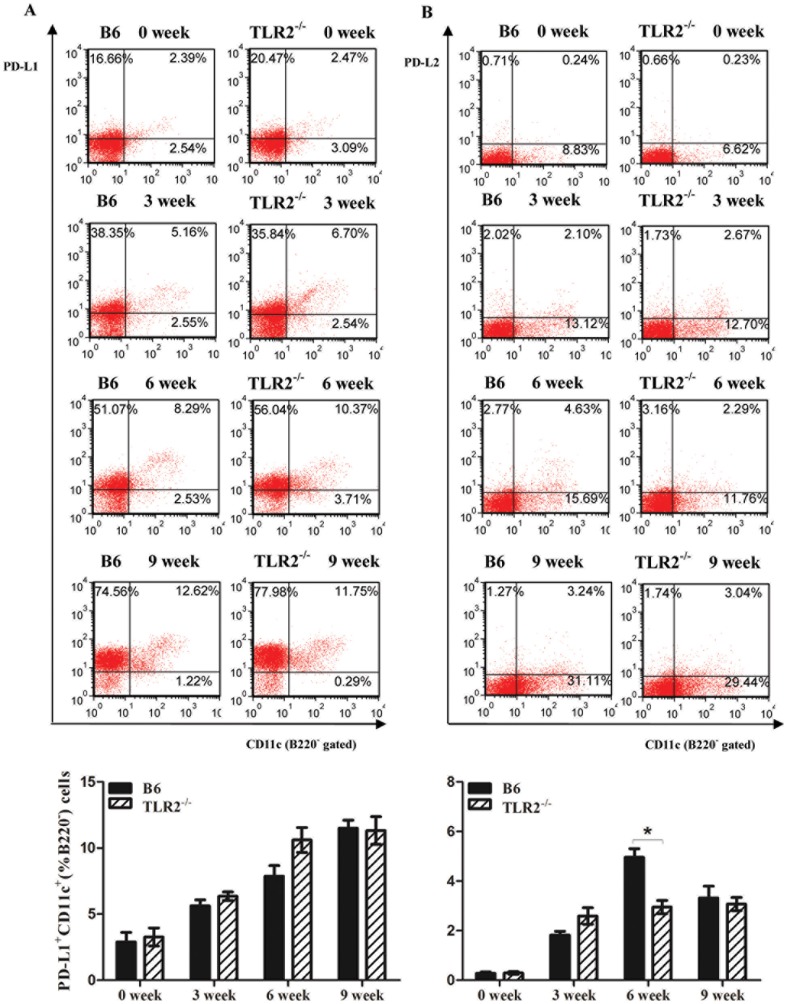
Dynamic changes of PD-L1(A) and PD-L2(B) expression on CD11c^+^ dendrtic cells from TLR2^−/−^ and B6 mice during the chronic infection with *Schistosoma japonicum* for 14 cercariae. Here was the result of one experiment (n = 6). Data are presented as the mean±SEM. *P<0.05.

Besides that persistent expression of TLR2 was observed on DC during chronic infection with *S. japonicum*, we would like to make sure if the co-inhibitory molecules, PD-L1 and PD-L2 on dendritic cells were involved in the immune regulation in this process. As shown in Figure 4, as well as TLR2 expression trend, the expression of PD-L1 and PD-L2 on CD11c^+^ cells was rapidly upregulated and maintained high levels as schistosome infection was continued. We found that in the course of schistosome infection, there was no significant difference on the expression of PD-L1 on CD11c^+^ cells between TLR2^−/−^ and B6 mice. But at 6 weeks after infection, the level of PD-L2 on CD11c^+^ cells from TLR2^−/−^ mice was significantly lower than that of B6 mice. It was indicated that the upregulation of PD-L2 expression on CD11c^+^ cells, especially after the appearance of a large number of eggs might partly depend on TLR2 expression.

#### The expression of PD-1 on CD4+T cells, not on CD8+T cells, was down-regulated in TLR2−/− mice after S. japonicum infection

**Figure 5:**
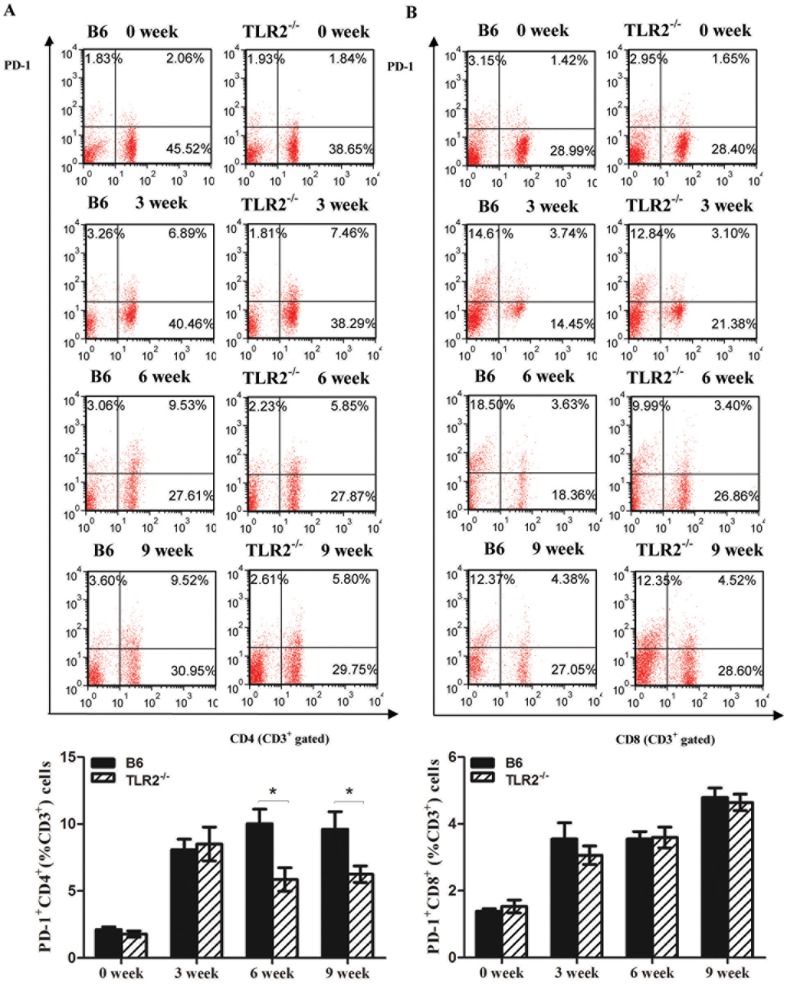
Dynamic changes of PD-1 expression on CD4^+^T(A) and CD8^+^T(B) cells from TLR2^−/−^ and B6 mice during the chronic infection with *Schistosoma japonicum* for 14 cercariae. Here was the result of one experiment (n = 6). Data are presented as the mean±SEM. *P<0.05.

Additionally, we also detected the expression of PD-1, the receptor of PD-L1 and PD-L2, on the T lymphocytes with or without TLR2 expression during schistosome infection. As shown in Figure 5, the expression of PD-1 on CD4^+^ or CD8^+^T cells was gradually increased after *in vivo* chronic *S. japonicum* infection in both TLR2^−/−^ and B6 mice. At 6 and 9 weeks after infection, the levels of PD-1 on CD4^+^T cells from TLR2^−/−^ mice were significantly lower than those of B6 mice. There were no significant differences on PD-1 expression from CD8^+^T cells between TLR2^−/−^ and B6 mice. After *S. japonicum* infection, TLR2 deficiency prevented effective T cell inhibition through attenuated PD-L2/PD-1 interaction, leading to enhance effector T cell responses.

#### Schistosome antigens, especially SEA, in vitro induced PD-L2 up-regulation on DCs was TLR2 dependent

**Figure 6:**
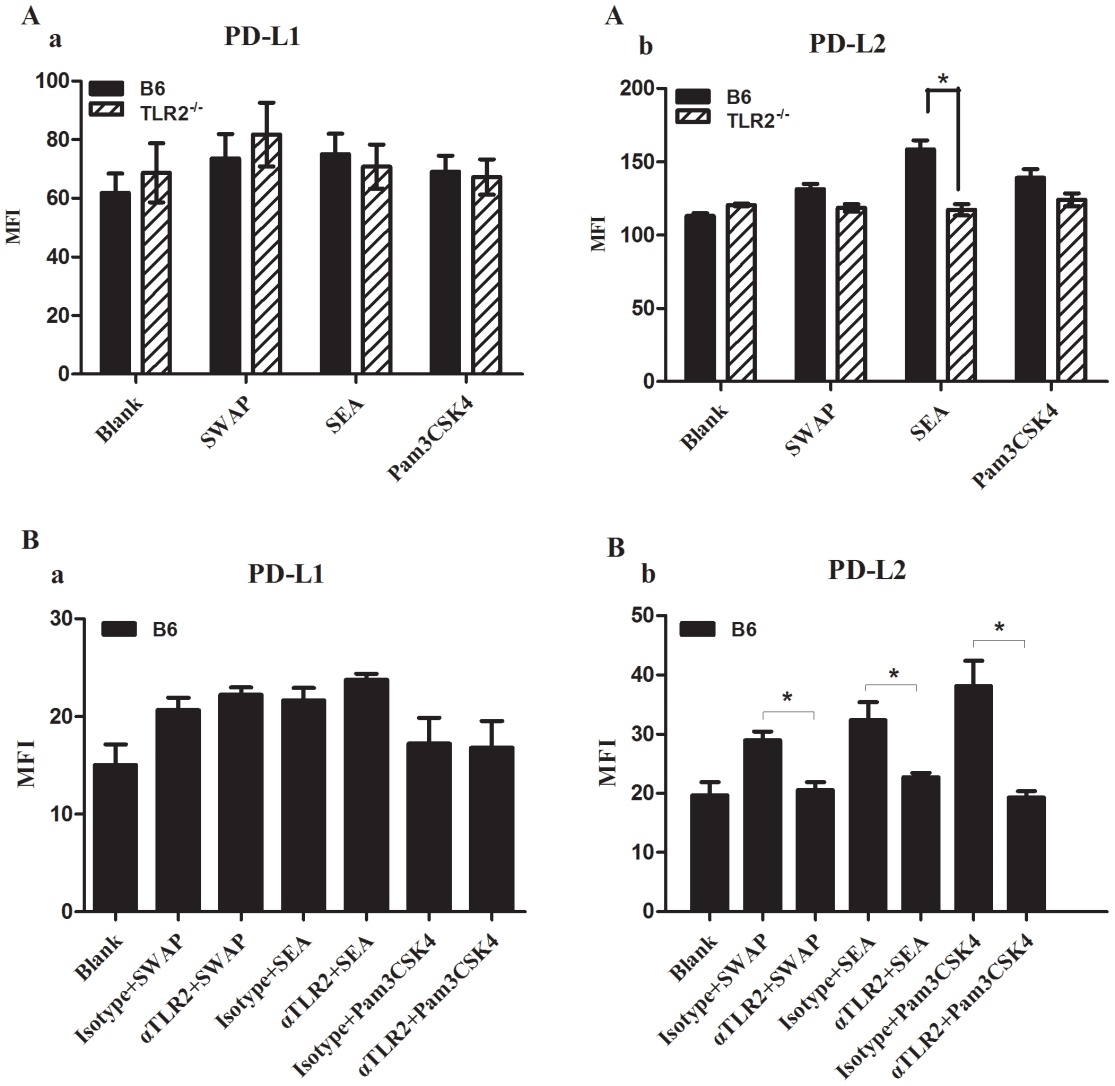
The expression of PD-L1 and PD-L2 on various schistosome antigens-treated BMDCs from TLR2^−/−^ and B6 mice or on B6 BMDCs with α-TLR2 antibody pre-blocking. BMDCs were stimulated with SWAP, SEA, Pam3CSK4 and a blank control. The expression of PD-L1(A a) and PD-L2(A b) on various schistosome antigens-treated BMDCs from TLR2^−/−^ and B6 mice. The expression of PD-L1(B a) and PD-L2(B b) on various schistosome antigens-treated B6 BMDCs with α-TLR2 antibody pre-blocking. Data are representative result of three independent experiments. Data are presented as the mean±SEM. *P<0.05.

To further define the interplay of TLR2 and PD-L2, we designed *in vitro* experiment to investigate the PD-L2 expression on bone marrow-derived dendritic cells (BMDCs) in the absence of TLR2 gene or the blockage of TLR2 expression using α-TLR2 blocking antibody. As shown in Figure 6A, we found that stimulation with schistosome specific antigens SWAP and SEA could induce a slight up-regulation of PD-L1 expression on the surface of BMDCs from B6 and TLR2^−/−^ mice. Moreover, there was no significant difference on the expression of PD-L1 with any stimulation between B6- and TLR2^−/−^ BMDCs. However, TLR2 deficiency on BMDCs resulted in a significantly decreased expression of PD-L2 with stimulation of SEA compared to B6 BMDCs.

Next, we explored a blocking α-TLR2 antibody to pre-treat BMDCs from normal mice to further observe the expression of PD-L1 and PD-L2 on BMDCs. As shown in Figure 6B, there was no significant difference on the expression of PD-L1 with stimulation of schistosome specific antigens between groups of blocking α-TLR2 antibody and isotype control. The expression of PD-L2 on BMDCs with a blocking α-TLR2 antibody prior to stimulation with SWAP, SEA or Pam3CSK4 was significantly decreased than that in groups of isotype control. These experiments suggested that PD-L2 expression depended to some extent on the expression of TLR2.

#### BMDCs with TLR2 deficiency or impaired expression of PD-L2 induced naïve CD4+T cells to express low level of IL-10 or high level of IFN-γ

**Figure 7:**
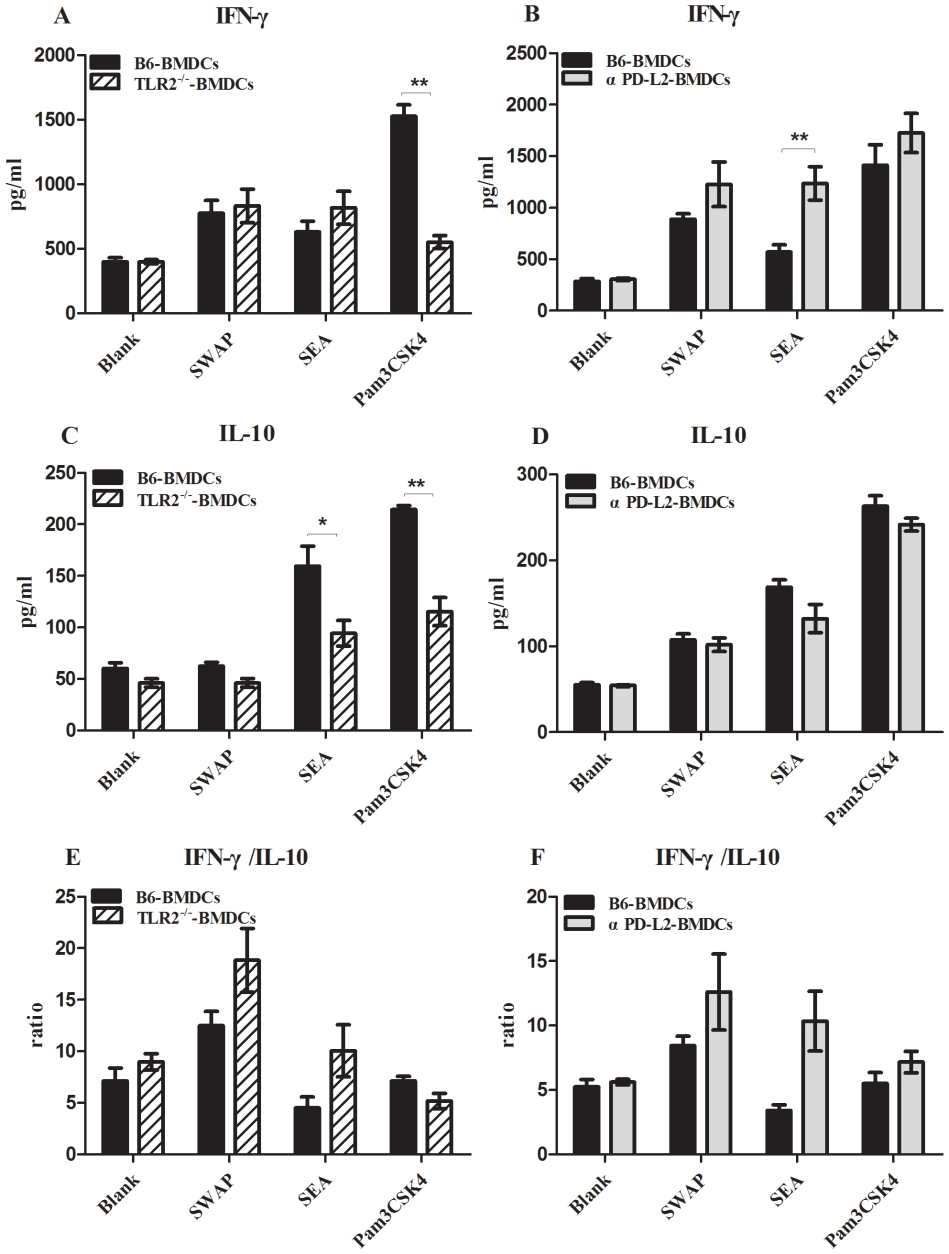
The level of IFN-γ and IL-10 produced by naïve CD4^+^T cells primed by TLR2^−/−^-BMDCs or B6 BMDCs pretreated with αPD-L2 antibody. BMDCs were stimulated with SWAP, SEA, Pam3CSK4 and a blank control. The level of IFN-γ(A), IL-10(C) and the ratio of IFN-γ/IL-10(E) produced by naïve CD4^+^T cells primed by TLR2^−/−^-BMDCs or B6 BMDCs. The level of IFN-γ(B), IL-10(D) and the ratio of IFN-γ/IL-10(F) produced by naïve CD4^+^T cells primed by B6 BMDCs pretreated with αPD-L2 antibody. Data are representative result of three independent experiments. Data are presented as the mean±SEM. *P<0.05, **P<0.01.

TLR2 deficiency could partly decrease the expression of PD-L2. We further tested if there would be the similar results of cytokine production from naïve CD4^+^T lymphocytes stimulated by BMDCs with the affected expression of TLR2 or PD-L2. As shown in Figure 7, the production of IFN-γ by CD4^+^T cells primed with SEA-stimulated TLR2^−/−^ BMDCs exhibited a trifling elevation compared with that from B6 mice, while IL-10 production by CD4^+^T cells primed with SEA-treated TLR2^−/−^ BMDCs was significantly reduced. In α PD-L2 antibody pre-blocking experiment, a significant increment on IFN-γ production with SEA stimulation was observed in the supernatant of co-culture with CD4^+^T cells and α PD-L2 antibody pretreated-BMDCs, while the ability of IL-10 production was slightly affected. Finally, we compared the ratio of IFN-γ/IL-10 production in CD4^+^T cells and found that this ratio in response to schistosome antigens stimulation was both higher in group of TLR2^−/−^ BMDCs or α PD-L2 antibody pretreated-BMDCs than that in normal BMDCs.

## Discussion

TLR2 is an important recognition molecule for multiple pathogens, including bacteria, viruses, fungi, and parasites. Here, we observed that TLR2 expression increased gradually on CD11c^+^ cells with the infection progression in *S. japonicum* infected B6 mice (Fig. 3). We further demonstrated that schistosome antigens (SWAP and SEA) up-regulated TLR2 expression on B6-BMDC *in vitro*, while SEA significantly elevated the *tlr2* mRNA levels in B6-BMDCs (data not shown). Our findings are consistent with the reearch by Correale [20] *et al* which showed TLR2 up-regulation on DCs and B cells exposed to *Schistosoma mansoni* SEA. Onquru [21] *et al* also described that human circulating B cells express surface TLR2 due to Schistosomiasis mansoni. All these suggested that schistosome antigens could induced TLR2 expression on DCs.

The wide array of TLR2 ligands includes molecules with diacyl and triacylglycerol moieties, proteins, polysaccharides [22], and a lipid fraction from *Schistosoma mansoni* eggs containing lysophosphatidylserine (lyso-PS) so on [7]. Some TLR2 ligands can induce IL-12p70 and stimulate Th1 response [23], [24], whereas some evidence demonstrate that TLR2-mediated signaling can also stimulate Th2 or T-regulatory response [23], [25]–[27]. Our study showed that TLR2 played a negative role in regulating host immune response during *Schistosoma japonicum* infection. At 6 weeks after *S. japonicum* infection, the percentage of CD4^+^T, CD8^+^T and Th1 cells in TLR2^−/−^ mice were significantly increased than that in B6 mice (Fig. 1A, 1B & 1C). Also, the mRNA levels of *il12* and *ifng* in CD4^+^ cells, and of *gzma*, *gzmb* and *fasl* in CD8^+^ cells were significantly elevated in TLR2^−/−^ mice compared to B6 mice (Fig. 2). We previously reported that compared to wild-type mice, TLR2^−/−^ mice produced elevated levels of IL-12 and IFN-γ in cultured pinna supernatants at 4 days after *S. japonicum* infection, with much higher IFN-γ and IL-12p70, and less IL-10 in the supernatant of spleen cells at the acute stage of infection [18]. These findings are in line with studies done by Layland *et al* in mice infected with *Schistosoma mansoni* [28]. There are also reports in other infectious mice models showed that TLR2 knockout enhanced T cell responses. Drennan *et al* showed that after infection with *Mycobacterium tuberculosis*, IFN-γ, TNF-α and IL-12p40 protein levels as well as CD4^+^ and CD8^+^ cells numbers increased in the lung of TLR2^−/−^ mice [29]. Furthermore, our study suggested that TLR2 deficiency mainly downregulated the PD-1 expression on CD4^+^T cells (Fig. 5), and then easily activate CD4^+^T cells. Other studies also showed that high PD-1 expression inhibits T cell activation [30], [31]. Taken together, these data suggest that during *Schistosoma japonicum* infection, an exaggerated T cells immune response takes place in the absence of TLR2 likely through down regulation of PD-1 expression.

We further explored the TLR2—PD-L2—PD1 pathway to illustrate the mechanisms involved in TLR2-induced immune suppression in schistosome infection. During *Schistosoma japonicum* infection, TLR2, PD-L1 and PD-L2 expression on DCs were persistently upregulated till 6 weeks after infection in wild-type mice (Fig. 3 & Fig. 4), whereas TLR2 deficiency results in decreased PD-L2 expression on DCs at 6 weeks after infection (Fig. 4B). Our *in vitro* experiments on SEA-treated TLR2^−/−^ DCs or α-TLR2 antibody pre-blocking DCs also verified that *Schistosoma japonicum* antigens, especially SEA could induce PD-L2 upregulation on DCs through TLR2-dependent pathway (Fig. 6A–b & 6B–b). Thus, we speculated that the regulatory function of TLR2 in schistosome infection is in part through selectively regulating PD-L2 expression. A study on *Mycobacterium avium* suggested that *Mycobacterium avium* induced high PD- L2expression on DCs, which was dependent on IL-10 production through the TLR2-p38 MAPK signaling pathway [32]. The MAPK family is composed of three major groups: p38, ERK1/2 and JNK1/2. Correale *et al* reported that DCs stimulation by SEA and TLR2 agonists induced increasing phosphorylation of the MAPK ERK1/2, neither stimulus showed an effect on p38 and JNK1/2 phosphorylation [20]. It is feasible that TLR2 activation on DCs is related to MAPK ERK1/2 signaling pathway in schistosome infection. There was a literature with the similar result as ours. Smith *et al* showed that *Schistosoma mansoni* worms induced anergy of T cells via up-regulation of PD-L1 on macrophages [33]. PD-L1 is constitutively expressed by a wide variety of immune cells and nonimmune cells in the presence of strong inflammatory signals, while PD-L2 was mainly expressed on antigen-presenting cells. Exposure of DCs and macrophages to some TLR ligands or Th2 cytokines (such as IL-4) can increase the expression of PD-L2, even primarily Th2 cells themselves activated in the presence of IL-4 can upregulate PD-L2 expression. It is not difficult to imagine that schistosome egg antigens, strong stimuli for Th2 response, can upregulate the PD-L2 expression from *in vivo* and *in vitro* evidence. Thus, in consideration of Smith's study on PD-L1 expression on macrophages and our study on PD-L2 expression on dendritic cells, we may infer that different TLRs signaling possibly mediates different pattern of co-inhibitory molecules expression on specific cell subsets.

PD-L1 and PD-L2, and their receptor, PD-1, form a co-inhibitory pathway that contributes to the negative regulation of T-lymphocyte activation [34], [35]. Generally thinking, PD-L2 is expressed at a lower level may favour PD-L1 as the primary binding ligand of PD-1, except for during Th2 responses when PD-L2 is upregulated. There are a number of studies showing that the PD-L2-PD1 pathway inhibited T cell proliferation and cytokine production [36], [37]. Terrazas *et al* found that *Taenia crassiceps* induced macrophages had the ability to suppress T cell proliferation *in vitro* in a cell contact-dependent manner via an increased expression of PD-L1 and PD-L2 [38]. Zhang *et al* found that PD-L2^−/−^ mice exhibited increased activation of CD4^+^ and CD8^+^ T cells *in vivo* when compared with WT animals [39]. In CBA/J mouse/*Schistosoma mansoni* chronic infection model, a significantly higher proportion of splenic CD11c^+^B220^−^DC express PD-L2, and a higher proportion of splenic CD4^+^T cells express PD-1 [40]. This result on *Schistosoma mansoni* was similar as ours. As *Schistosoma japonicum* infection progressing PD-1 expression was significantly up-regulated on both CD4^+^T cells or on CD8^+^T cell, however, the absence of TLR2 *in vivo* mainly affected PD-1 expression on CD4^+^T cells, not on CD8^+^T cells. Gaddis *et al* reported that up-regulation of PD-1 on CD4^+^T cells was partially dependent on IL-10, while IL-10 production was dependent on TLR2/1 signaling in *Porphyromonas gingivalis* infection [41]. Our previous studies suggested that TLR2 deficency led to attenuated IL-10 production in *Schistosoma japonicum* infection [19], which might help down-modulate PD-1 expression on CD4^+^T cells.

In conclusion, we deduced that TLR2 signaling might orchestrate the up-regulation of PD-L2 on dendritic cells, which binds to PD-1 on T cells (mainly on CD4^+^T cells), to help inhibit T cell-mediated immune response, especialy after a large number of eggs appearing during schistosome infection. The relevant molecular mechanisms on TLR2 regulating PD-L2 expression on DCs will be further investigated. Our study will contribute to understand the mechanisms of immune downregulation in many chronic infections, viewed from innate immunity point.

## Methods

### Mice and parasites

Six- to eight-week-old female TLR2-deficient (TLR2^−/−^) mice, as well as the wild-type (WT) control C57BL/6J (B6) mice were purchased from Model Animal Research Center of Nanjing University (Nanjing, China). TLR2^−/−^ mice on a B6 background were validated and bred by homozygotes. All mice were maintained and bred under specific pathogen-free conditions. All experiments were undertaken with the approval of Nanjing Medical University Animal Ethics Committee.

*Schistosoma japonicum* (*S. japonicum*, a Chinese mainland strain) cercariae were maintained in *Oncomelania hupensis* snails as the intermediate host, which were purchased from Jiangsu Institute of Parasitic Disease (Wuxi, China).

The soluble worm antigen (SWAP) and the soluble egg antigen (SEA) were prepared as before [19]. The concentrations of antigens were assayed using a BCA Protein Assay Kit (Pierce, Rockford, IL, USA). The concentrations of endotoxin in these antigens were below 0.03 EU/mL assayed by a timed gel endotoxin detection kit (Sigma, St. Louis, MO, USA).

### *In vivo* acute infection with Schistosoma japonicum

#### Infection of mice

TLR2^−/−^ and B6 mice were infected percutaneously with 40 *S. japonicum* cercariae. There were eight mice in each group including uninfected and infected mice. At six weeks post-infection, all mice were sacrificed. Two independent experiments were performed.

#### Isolation of splenocytes and calculation of the percentage of some immune cell subsets by flow cytometry

Spleens were aseptically removed when uninfected mice and 6 weeks-*S. japonicum* infected mice were sacrificed. Spleen cells were prepared by gently forcing spleen tissue through a fine nylon net into incomplete RPMI-1640 medium (Gibco-Invitrogen, Grand Island, NY) which containing 100 U/ml penicillin-streptomycin (Gibco-Invitrogen). After removal of erythrocytes, the cells were resuspended in complete RPMI-1640 medium which containing incomplete RPMI-1640 and 10% fetal bovine serum (Gibco-Invitrogen).

1.0×10^6^ splenocytes were resuspended in staining buffer (PBS/2% fetal calf serum) and stained with the following antibodies for 20 minutes at 4°C in the dark: APC anti-mouseCD3e, FITC anti-mouse CD4 and FITC anti-mouse CD8a (eBioscience, San Diego, CA). Antibody-treated cells were washed twice with staining buffer.

For detection of Th1, Th2, Tc1 or Tc2 cells, 2.0×10^6^ splenocytes were stimulated with 25 ng/ml PMA (Enzo Life Science, New York, USA) and 1 µg/mL ionomycin (Enzo Life Science) in the presence of 10 µg/ml Brefeldin-A (Enzo Life Science) for 6 h at 37°C in 5% CO_2_. After 6 h, the cells were collected and surface stained with APC anti-mouse CD3e (eBioscience) and FITC anti-mouse CD4 (or CD8) (eBioscience). Subsequently, the cells were washed, fixed and permeabilized with Cytofix/Cytoperm buffer (eBioscience) and intracellularly stained with PE conjugated antibodies against IFN-γ or IL-4 (or isotype IgG2a control antibody) (eBioscience), respectively. Stained cells were collected by BD FACScalibur. Cells were gated on the CD3^+^CD4^+^ (or CD3^+^CD8^+^) population for analysis of Th1 or Th2 (Tc1 or Tc2) cells. According to isotype IgG2a control antibody, we defined the quadrants to evaluate the intracelluar level of IFN-γ or IL-4.

Cells were detected by flow cytometry (Becton Dickinson). All data were analyzed using CellQuest software (Becton Dickinson).

#### Purification of CD4+ or CD8+ cell subsets by magnetic-activated cell sorting (MACS)

Splenocytes of TLR2^−/−^ and B6 mice at 6 weeks post-infection were harvested. CD4^+^ or CD8^+^ cells of each mouse were purified by positive using magnetic sorting (MACS; Mini Macs, Miltenyi Biotec). Purified CD4^+^ or CD8^+^ cells were used for the quantitative real-time PCR analysis.

#### Quantitative real-time PCR (qRT-PCR)

Purified CD4^+^ or CD8^+^ cells were lysed and total RNA was extracted using TRIzol reagent (Invitrogen Life Technologies, Carlsbad, USA) and reverse-transcribed to cDNA using the PrimeScript RT reagent kit (Takara, Otsu, Japan). cDNA was amplified with SYBR Green Master (Roche, Basel, Switzerland) in a 7300 Real-time PCR System (Applied Biosystems, Foster City, USA). Primers specific for β-actin, IL-12 (*il12*), IFN-γ (*ifng*), IL-10 (*il10*), granzyme A (*gzma*), granzyme B (*gzmb*) and Fas Ligand (*fasl*) are shown in Table 1. Real-time PCR was run in triplicates in a volume of 20 µl containing 10 µl of SYBR Green PCR Master, 300 nM of each primer, and 50 ng cDNA. Reaction conditions were as described for the SYBR Green kit and the cycling protocol was as follows: 50°C for 2 min, 95°C for 10 min, 40 cycles of 95°C for 15 s, 60°C for 1 min. The housekeeping gene β-actin was used as an internal control. Quantitations of relative differences in expression were finally calculated using the comparative 2^−ΔΔCt^ method.

**Table 1:** Primers and annealing temperatures used for the amplification of each target gene.

Gene	GenBank	Annealing temperature (°C)	Primer (5′→3′)
*β-actin*	NM 007393	68	F:5′CCTCTATGCCAACACAGTGC3 R:5′GTACTCCTGCTTGCTGATCC3
*il12*	NM 008351.2	59	F:5′TGATGATGACCCTGTGCCTT R:5′CTGCTGATGGTTGTGATTCTGA
*ifng*	NM 008337.3	59	F:5′AGCAACAACATAAGCGTCAT R:5′CCTCAAACTTGGCAATACTC
*il10*	NM 010548.2	59	F:5′CAACATACTGCTAACCGACTC R:5′CATTCATGGCCTTGTAGACAC
*gzma*	NM 010370.2	68	F:5′TGTGAAACCAGGAACCAGATG R:5′GGTGATGCCTCGCAAAATA
*gzmb*	NM 013542.2	68	F:5′TGCTCTGATTACCCATCGTCC R:5′GCCAGTCTTTGCAGTCCTTTATT
*fasl*	NM 010177.3	68	F:5′GGTTCTGGTGGCTCTGGTT R:5′ACTTTAAGGCTTTGGTTGGTG
*tlr2*	NM 011905.3	59	F:5′GAAACCTCAGACAAAGCGTC R:5′GCTTTTCATGGCTGCTGTGA

### *In vivo* chronic infection with Schistosoma japonicum

#### Infection of mice

TLR2^−/−^ and B6 mice were infected with 14 *S. japonicum* cercariae through the abdominal skin. At 0, 3, 6 and 9 weeks post-infection, six mice each group were randomly sacrificed for further study. Spleens were aseptically removed when uninfected mice and 3, 6 and 9 weeks-*S. japonicum* infected mice were sacrificed. The splenocytes from TLR2^−/−^ and B6 mice were prepared as described above.

#### Flow cytometry analysis

1.0×10^6^ splenocytes were resuspended in staining buffer and stained with the following antibodies for 20 minutes at 4°C in the dark: APC anti-mouse CD11c, PE anti-mouse TLR2, PE anti-mouse PD-L1, PE anti-mouse PD-L2, FITC anti-mouse B220, APC anti-mouse CD3e, FITC anti-mouse CD4, FITC anti-mouse CD8a and PE anti-mouse PD-1 (eBioscience). Antibody-treated cells were washed twice with staining buffer. Cells were detected by flow cytometry (Becton Dickinson). All data were analyzed using CellQuest software (Becton Dickinson).

#### Purification of CD11c+ subsets by magnetic-activated cell sorting (MACS)

The splenocytes harvested from B6 mice at 3, 6 and 9 weeks post-infection. CD11c^+^ cells from a mouse spleen cell suspension were purified by positive using magnetic sorting (Miltenyi Biotec). Purified CD11c^+^ cells were used for the quantitative real-time PCR analysis.

#### Quantitative real-time PCR (qRT-PCR)

Purified CD11c^+^ cells from B6 mice were lysed and total RNA was extracted using TRIzol reagent (Invitrogen Life Technologies) and reverse-transcribed to cDNA using the PrimeScript RT reagent kit (Takara). Primers specific for β-actin and TLR2 (*tlr2*) are shown in Table 1. Real-time PCR was run in triplicates in a volume of 20 µl containing 10 µl of SYBR Green PCR Master, 300 nM of each primer, and 50 ng cDNA. Reaction conditions were as described above.

### *In vitro* experiments

#### Generation of bone marrow-derived dendritic cells (BMDCs) and stimulations

Bone marrow cells were collected aseptically from the femurs and tibias of TLR2^−/−^ and B6 mice, processed with red blood cell lysing buffer. Cells (1×10^6^) were cultured in 24-well plates (Corning Incorporated) for 7 days in 1 ml of complete DC medium consisting of RPMI-1640 medium, 100 U/ml penicillin-streptomycin and 10% fetal bovine serum (Gibco-Invitrogen), supplemented with 10 ng/ml recombinant murine granulocyte-macrophage colony-stimulating factor (GM-CSF; PeproTech, Rocky Hill, USA) and 5 ng/ml recombinant murine interleukin-4 (IL-4; PeproTech). On days 3 and 5 after the start of the culture, 0.5 ml of medium was removed and replaced with fresh medium. On day seven, non-adherent cells were collected and purified by CD11c^+^ positive selection using magnetic sorting (Miltenyi Biotec).

1.0×10^6^ BMDCs derived from TLR2^−/−^ and B6 mice were pulsed with Pam3CSK4 (5 µg/ml; InvivoGen, San Diego, USA), SWAP (50 µg/ml) or SEA (50 µg/ml) in triplicate. Unstimulated BMDCs served as blank controls. After 24 hours, the harvested BMDCs were washed twice with PBS, and used for further study. Three independent experiments were performed.

In some experiments, BMDCs derived from B6 mice were incubated with or without a blocking monoclonal antibody against 10 µg/ml TLR2 (T2.5; BioLegend, San Diego, USA), 2.5 µg/ml PD-L2 (TY25; eBioscience) or isotype control for 1 h prior to treatment with various antigens. Then, α-TLR2 antibody-blocked BMDCs were used to detect the expression of PD-L1 and PD-L2 on the surface by flow cytometry. α-PD-L2 antibody-blocked BMDCs were co-incubated with naïve T cells for assaying the levels of cytokines in the supernatant.

#### Flow cytometry analysis

Antigen-pulsed BMDCs were resuspended in staining buffer and stained with the following antibodies for 20 minutes at 4°C in the dark: PE anti-mouse PD-L1 and PE anti-mouse PD-L2 (eBioscience). Antibody-treated cells were washed twice with staining buffer. Cells were detected by flow cytometry (Becton Dickinson FACScalibur). All data were analyzed using CellQuest software (Becton Dickinson).

#### Antigen-pulsed BMDCs with naïve T cells co-culture

Antigen-pulsed BMDCs from TLR2^−/−^ and B6 mice, and BMDCs pre-blocked with αPD-L2 antibody were harvested. Naïve CD4^+^ T cells were purified from B6 mice by positive selection using magnetic sorting (Miltenyi Biotec). BMDCs (2×10^4^) were added to CD4^+^ T cells (2×10^5^ cells per well) in round-bottom 96-well tissue culture plates (Corning Incorporated). After 72 hours, the culture supernatants were collected and the concentrations of IFN-γ and IL-10 (eBioscience) were measured by ELISA according to the instructions provided by the manufacturer. Three independent experiments were performed.

### Statistical analysis

The data were presented as the mean ± SEM. Significance was tested using a Student's *t*-test for paired observations. Significant values were indicated as follows: * *P*<0.05, **P<0.01.
